# Left atrial shortening fraction to predict fetal cardiac abnormalities and dysfunction in gestational diabetes mellitus

**DOI:** 10.3389/fcvm.2022.1026587

**Published:** 2022-12-16

**Authors:** Yu-Qing Peng, Xuan Qiu, Li Wang, Xin Li, Xiao-Yan Huo

**Affiliations:** ^1^Department of Ultrasonic, Chinese PLA General Hospital-Sixth Medical Center, Beijing, China; ^2^The Second School of Clinical Medicine, Southern Medical University, Guangzhou, China

**Keywords:** gestational diabetes mellitus, fetal echocardiography, septal hypertrophy, cardiac dysfunction, left atrial shortening fraction

## Abstract

**Objective:**

To evaluate the diagnostic efficiency of left atrial shortening fraction (LASF) in the detection of fetal cardiac abnormalities and dysfunction in patients with gestational diabetes mellitus (GDM).

**Methods:**

In this study, we enrolled 256 pregnant women and divided them into GDM group (*n* = 156) and control group (*n* = 100). Fetal echocardiography was performed at 24–28 weeks of gestation to measure the LASF and interventricular septum (IVS) thickness. Based on IVS thickness, the GDM group was subdivided into the septal hypertrophy group (GDM I, *n* = 62) and non-septal hypertrophy group (GDM II, *n* = 94). LASF and IVS thickness were compared between the GDM and control groups and between GDM I and GDM II groups. Receiver operating characteristic (ROC) analysis was performed to determine the diagnostic accuracy of LASF in predicting septal hypertrophy.

**Results:**

The GDM group had a larger IVS thickness (*P* < 0.05) but a lower LASF level (*P* < 0.001) than those of the control group. GDM I group had significantly lower LASF level than that in the GDM II group (*P* < 0.001). At 38.41% as the cutoff value, the LASF can predict septal hypertrophy with diagnostic sensitivity and specificity of 96.7% and 65.2%, respectively.

**Conclusion:**

Fetal GDM are more likely to induce septal hypertrophy and ventricular dysfunction. LASF is a good indicator of septal hypertrophy or early diastolic dysfunction without septal hypertrophy.

## 1 Introduction

International Diabetes Federation estimates that 16.2% of women with live births had hyperglycemia in pregnancy (1). The incidence of congenital cardiac malformations in the offspring of diabetic mothers (3–6%) is considerably higher than in the normal population (2). Septal hypertrophy and cardiac dysfunction are often found in fetuses with gestational diabetes mellitus (GDM), and diastolic dysfunction develops earlier than systolic dysfunction (3, 4).

Fetal echocardiography is the benchmark for diagnosing heart structural and functional abnormalities in fetuses with GDM. The myocardial performance index (MPI), mitral valve E/A ratio, and tricuspid valve E/A ratio are intensively used for indicating fetal cardiac function (5, 6). Although MPI measurement is not affected by ventricular geometry, cardiac load, nor heart rate, it is more time-consuming than atrioventricular valve E/A ratio. Moreover, it is difficult to distinguish between systolic dysfunction and diastolic dysfunction when MPI is abnormal. Mohsin M reported that left atrial shortening fraction (LASF) could be used to estimate early cardiac dysfunctional without septal hypertrophy in GDM fetuses (7). This study aims to investigate the diagnostic efficacy for LASF in predicting cardiac abnormalities and ventricular dysfunction in fetuses with GDM.

## 2 Materials and methods

### 2.1 Subjects and grouping

This retrospective study enrolled 256 singleton pregnant women at gestational ages of 24–28 weeks who underwent echocardiography at the Sixth Medical Center of PLA General Hospital between January 2020 and January 2022. The study was approved by the PLA General Hospital ethics committee (HZKY-PJ-2022-6). Among them, 156 women (age, 20–42 years; mean age, 33.27 ± 3.97 years) were diagnosed with GDM. Their gestational weeks ranged from 24th to 28th with a mean of (26.65 ± 1.14). The control group consisted of 100 normal pregnant women aged 20–40 years (average age, 32.06 ± 3.16 years), gestational age of 24th–28th weeks and mean gestational age of (26.43 ± 0.53) weeks. Age, height, weight, body mass index (BMI), and gestational age were collected and recorded (Table 1).

**TABLE 1:** Comparison of general clinical data between two groups.

Variable	GDM group (*n* = 156)	Controlgroup(*n* = 100)	*t*	*P*
Maternal age (year)	33.27 ± 3.97	32.06 ± 3.16	1.438	0.154
Height (cm)	163.62 ± 5.47	167.32 ± 4.81	-0.346	0.606
Weight (kg)	66.62 ± 9.95	63.36 ± 6.64	1.620	0.109
BMI (kg/m^2^)	24.86 ± 2.29	21.71 ± 1.79	2.057	0.023
EGA (week)	26.65 ± 1.14	26.43 ± 0.53	0.952	0.344

BMI, body mass index; EGA, estimated gestational age.

In this study, the inclusion criteria were as follows: all pregnant women were diagnosed with GDM defined by the World Health Organization in 2010 (8), Normal blood glucose level (control group), singleton pregnancy, and sound mental health and can cooperate with the examinations.

The exclusion criteria were as follows: other pregnancy complications, such as pregnancy-induced hypertension and intrahepatic cholestasis of pregnancy; thyroid disease, rheumatic immune disease, hypertension, or pregestational diabetes; congenital heart disease or arrhythmia; twins or multiple pregnancy; mental disorders or cognitive disorders; and incomplete clinical data and incomplete echo parameters.

### 2.2 Instruments

GE Voluson 8 ultrasound device equipped with RAB4-8-D probe (4–8 HMz) was used.

### 2.3 Fetal echocardiography protocol

#### 2.3.1 Parameters for fetal cardiac structure and function estimate

1. Left ventricular diastolic functional parameters: mitral valve E/A ratio, LASF;

2. Right ventricular diastolic functional parameters: tricuspid valve E/A ratio, DV-PI;

3. Cardiac structural parameters: Interventricular septal thickness at end-diastolic (IVSd).

#### 2.3.2 Methods for parameters measurement

All women with gestational age of 24th–28th weeks underwent echocardiography. According to the echocardiography guidelines of American Society of Echocardiography (ASE) (9), pregnant women were placed in the supine position, and blood flow velocity of atrioventricular valve in early diastole (E peak) and late diastole (A peak) was measured to calculate E/A ratio. The blood flow spectrum in the ductus venous was acquired at the entrance of DV on the coronal section of the epigastrium. Ductus venosus pulsatility index (DV-PI) value was automatically recorded with a high-quality DV spectrum image acquired continuously within 3–5 cardiac cycles. IVS thickness at end-diastolic (IVSd) was recorded with M-mode in the long axis view. Then, the M-mode sampling line was moved to the left atrium, kept away from oval foremen, and the left atrial diameter at end-systolic (LAIDs) and end-diastolic (LAIDd) were measured successively, followed by LASF = (LAIDs-LAIDd)/LAIDs. All parameters were measured three times and averaged.

### 2.4 Statistical analysis

SPSS 26.0 software was used for statistical analysis. Measurement indicators were expressed as mean ± standard deviation (± s). Independent sample *T*-test was performed between GDM group and control group and between GDM I group and GDM II group. Receiver operating characteristic (ROC) analysis was applied to verify the diagnostic accuracy in predicting septal hypertrophy. *P*-value less than 0.05 is considered statistically significant.

## 3 Results

### 3.1 Comparison of general clinical data between the GDM group and the control group

Age, height, weight, and gestational age were not significantly different between the two groups (*P* > 0.05), except for BMI, which is higher in the GDM group than that in the control group (*P* < 0.05) (Table 1).

### 3.2 Comparison of fetal echocardiography parameters between the GDM group and the control group

**FIGURE 1:**
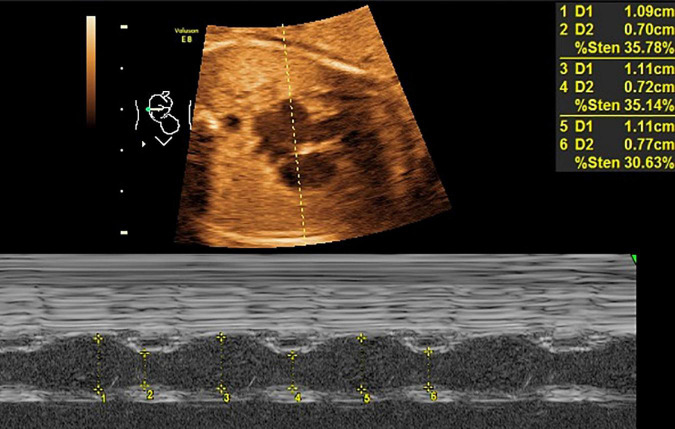
LASF in GDM fetuses at gestational age 26 weeks + 3 days. LASF, left atrial shortening fraction; GDM, gestational diabetes mellitus.

**FIGURE 2:**
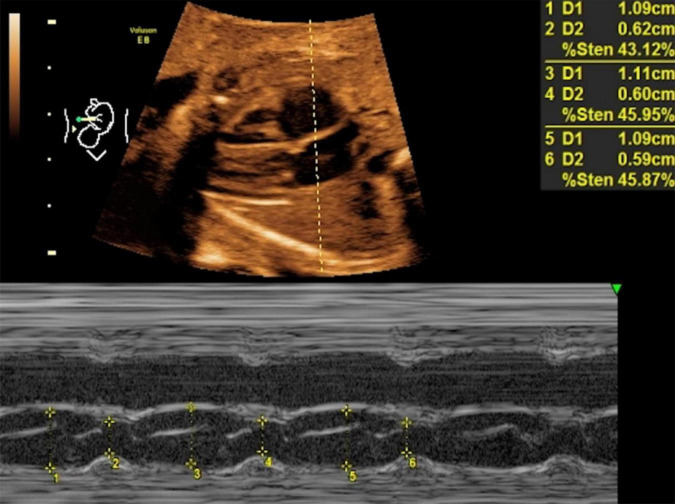
LASF in control fetuses at gestational age 26 weeks + 3 days. LASF, left atrial shortening fraction.

Mitral valve E/A ratio and tricuspid valve E/A ratio were not significantly different between the two groups (*P* > 0.05). GDM group showed significantly thicker IVSd (*P* < 0.001), higher DV-PI (*P* < 0.05), and significantly lower LASF than those in the control group (*P* < 0.001) (Figures 1, 2 and Table 2).

**TABLE 2:** Comparison of echocardiography parameters between two groups.

Variable	GDM group (*n* = 156)	Controlgroup(*n* = 100)	*t*	*P*
Mitral E/A ratio	0.61 ± 0.11	0.60 ± 0.10	0.146	0.884
Tricuspid E/A ratio	0.63 ± 0.09	0.63 ± 0.10	–0.075	0.941
IVSd (mm)	2.76 ± 0.55	2.31 ± 0.41	3.903	<0.001
LASF (%)	42.73 ± 6.04	51.17 ± 5.43	–4.058	<0.001
DV-PI	0.72 ± 0.26	0.56 ± 0.17	3.208	0.002

IVSd, interventricular septal thickness at end-diastolic; LASF, left atrial shortening fraction; DV-PI, ductus venosus-pulsatility index.

### 3.3 Comparison of general clinical data between the GDM I group and the GDM II group

Age, height, weight, gestational age and BMI were not significantly different between the two groups (*P* > 0.05) (Table 3).

**TABLE 3:** Comparison of general clinical data between GDM I group and GDM II group.

Variable	GDM I group (*n* = 62)	GDMIIgroup(*n* = 94)	*t*	*P*
Maternal age (year)	33.57 ± 4.02	32.53 ± 3.87	0.849	0.400
Height (cm)	161.3 ± 4.37	164.54 ± 5.65	-1.967	0.055
Weight (kg)	67.88 ± 5.60	64.91 ± 4.92	1.724	0.097
BMI (kg/m^2^)	25.77 ± 3.08	23.98 ± 2.82	1.827	0.063
EGA (week)	26.57 ± 1.23	26.71 ± 0.93	-0.416	0.679

GDM I group, GDM fetuses with septal hypertrophy; GDM II group, GDM fetuses without septal hypertrophy.

### 3.4 Comparison of fetal cardiac functional parameters between GDM I group and GDM II group

Septal hypertrophy was detected in 62 patients in the GDM group. Only LASF was significantly lower than that in non-septal hypertrophy group (*p* < 0.001). Atrioventricular valve E/A ratio and DV-PI were not significantly different between the two groups (*P* > 0.05) (Table 4).

**TABLE 4:** Comparison of cardiac functional parameters between GDM I group and GDM II group.

Variable	GDM I group (*n* = 62)	GDMIIgroup(*n* = 94)	*t*	*P*
Mitral E/A ratio	0.60 ± 0.12	0.65 ± 0.09	–1.134	0.187
Tricuspid E/A ratio	0.62 ± 0.10	0.65 ± 0.08	–1.194	0.238
LASF (%)	36.71 ± 5.32	44.32 ± 4.09	–4.973	<0.001
DV-PI	0.75 ± 0.19	0.68 ± 0.28	1.163	0.211

### 3.5 Diagnostic efficacy of LASF to predict GDM fetal septal hypertrophy with ROC curve analysis

**FIGURE 3:**
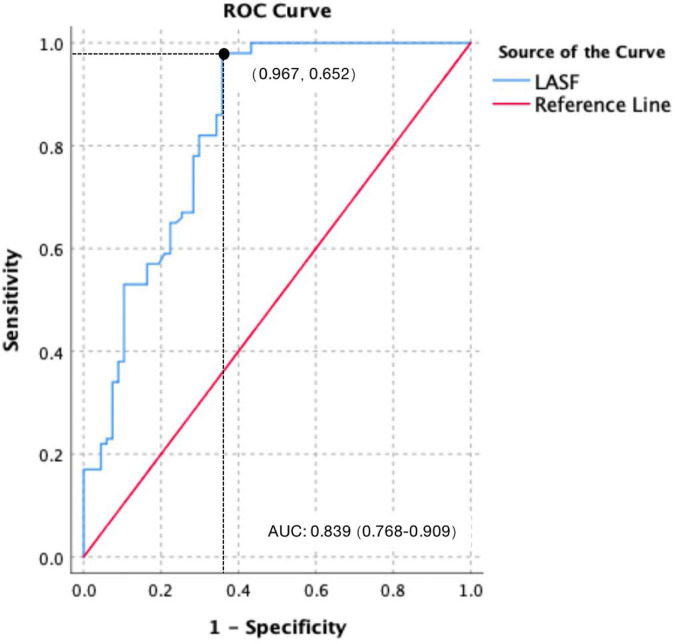
Receiver operating characteristic (ROC) curve of LASF to predict GDM fetal septal hypertrophy.

Using 38.41% as the cutoff value, the diagnostic sensitivity and specificity for LASF to predict GDM fetal septal hypertrophy were 96.7% and 65.2%, respectively. The area under the curve (AUC) and 95% confidence interval (CI) were 0.839 and 0.768–0.909, respectively (Figure 3).

## 4 Discussion

Fetuses with GDM are more likely to induce septal hypertrophy combined with cardiac dysfunction than non-GDM ones (10–12). Diastolic dysfunction comes earlier than systolic dysfunction (13, 14). IVS thickness can be used as an independent predictor of macrosomia (15). In this study, IVSd and DV-PI in GDM group were both higher than those in the control group, and LASF was significantly lower than that in the control group, which confirmed that septal hypertrophy combined with diastolic dysfunction were accompanied with GDM fetuses. IVSd, LASF, and DV-PI can be used to detect cardiac abnormalities and dysfunction, respectively.

Although E/A ratio was the most common diastolic function parameter (16), there were no significant differences in atrioventricular valve E/A ratio between GDM group and the control group, GDM I group and GDM II group. This result is consistent with the conclusion from Ren (17). This finding is not consistent with that reported by Ghaderian (18) and Hou (19) owing to several reasons. First, fetal E/A measurements are markedly affected by fetal respiration, fetal movement, and other factors. The measurement of E/A might be limited in cases of a very high fetal heart rate. It is difficult to distinguish E wave and A wave in case that fetal heart rate is too fast. In addition, the E/A ratio, which tend to decrease during pregnancy with GDM, might gradually increase with increasing gestational age.

In this study, LASF in the GDM fetuses with septal hypertrophy was significantly lower than that without septal hypertrophy, which was consistent with the results of Mohsin and Zielinsky (7, 20). Using 38.41% as the cutoff value, the diagnostic sensitivity and specificity for LASF to predict septal hypertrophy were 96.7% and 65.2%, respectively. The AUC and 95% CI were 0.839 and 0.768–0.909. Septal hypertrophy could lead to a decreased ventricular compliance (21), which causes a decreased blood flow from the mitral valve to left ventricular in late diastole. A greater amount of blood flow remained in the left atrium come to a decreased LASF consequently. Therefore, LASF is negatively correlated with interventricular septum thickness. Moreover, LASF measurement is easy and repeatable, which makes LASF a good index to predict septal hypertrophy and to detect the early diastolic dysfunction without septal hypertrophy.

In GDM fetuses with septal hypertrophy, increased right atrial pressure is associated with right ventricular dysfunction (22). DV blood flow spectrum showed a decreased flow velocity and an increased DV-PI in GDM fetuses (23, 24). In this study, DV-PI in the GDM group was higher than that in the control group, indicating that DV-PI had a certain efficacy to diagnosis right heart dysfunction. However, there was no difference in DV-PI between GDM I group and GDM II group, it seemed that this result from our study was not accord with the conclusion from Deng XD (25). The reason for the inconsistency between this conclusion and the previous DV-PI relevant conclusion maybe includes:

The study had limitations. First, even though all fetal echocardiographic examinations were independently performed by a senior physician and all examination parameters were aimed to be measured on the standard ultrasound section, man-made measurement error was inevitable. A standard DV spectrum was difficult to obtain continuously to automatically calculate DV-PI during middle and later terms of pregnancy, especially for the pregnant women with high BMI; Only 156 subjects comprised the GDM group, thereby limiting our findings.

Fetuses with GDM have septal hypertrophy and ventricular diastolic dysfunction. LASF and DV-PI can be used to detect left and right ventricular diastolic dysfunction, respectively. LASF is a prominent echo parameter to diagnose fetuses’ cardiac abnormalities and dysfunction.

## Data availability statement

The raw data supporting the conclusions of this article will be made available by the authors, without undue reservation.

## Ethics statement

The studies involving human participants were reviewed and approved by the Ethics Committee, The Sixth Center of PLA General Hospital (HZKY-PJ-2022-6). Written informed consent for participation was not required for this study in accordance with the national legislation and the institutional requirements.

## Author contributions

Y-QP contributed to the conceptualization. XQ contributed to the methodology. LW contributed to the data collection and statistical analysis. XL and X-YH contributed to the reviewing and editing of the manuscript. All authors have read and approved the final manuscript.
